# Inhibition of Herpes Simplex Viruses, Types 1 and 2, by Ginsenoside 20(S)-Rg3

**DOI:** 10.4014/jmb.1908.08047

**Published:** 2019-11-06

**Authors:** Stephen M. Wright, Elliot Altman

**Affiliations:** Department of Biology and the Tennessee Center for Botanical Medicine Research, Middle Tennessee State University, TN 37132, USA

**Keywords:** Ginsenoside Rg3, *Panax ginseng*, herpes simplex viruses, valacyclovir, virus inhibition

## Abstract

Infections by herpes simplex viruses have an immense impact on humans, ranging from selflimiting, benign illness to serious, life-threatening diseases. While nucleoside analog drugs are available, resistance has been increasing and currently no vaccine exists. Ginsenosides derived from *Panax ginseng* have been documented to inhibit several viruses and bolster immune defenses. This study evaluated 12 of the most relevant ginsenosides from *P. ginseng* for toxicities and inhibition of herpes simplex viruses types 1 and 2 in Vero cells. The effects of test compounds and virus infection were determined using a PrestoBlue cell viability assay. Time course studies were also conducted to better understand at what points the virus life cycle was affected. Non-toxic concentrations of the ginsenosides were determined and ranged from 12.5 μM to greater than 100 μM. Ginsenoside 20(S)-Rg3 demonstrated the greatest inhibitory effect and was active against both HSV-1 and HSV-2 with an IC_50_ of approximately 35 μM. The most dramatic inhibition—over 100% compared to controls—occurred when the virus was exposed to 20(S)-Rg3 for 4 h prior to being added to cells. 20(S)-Rg3 holds promise as a potential chemotherapeutic agent against herpes simplex viruses and, when used together with valacyclovir, may prevent increased resistance to drugs.

## Introduction

The *Herpesviridae* family is comprised of three subfamilies designated alpha, beta and gamma. Herpes simplex viruses types 1 and 2 (HSV-1 and HSV-2) are included within the alpha herpesviruses. HSV-1 is most commonly associated with oral infection and is reportedly present in nearly 70%of humans [1]. Other syndromes frequently associated with HSV-1 infection include keratitis and herpes gladiatorum, or skin disease in wrestlers. HSV-2 is more frequently associated with genital infection and may result in neonatal disease. In addition to these mucocutaneous infections, more severe, life-threatening disease may occur. In the United States, herpes simplex viruses are responsible for nearly 20% of encephalitis cases [2]. As reviewed [3], there is evidence for an association between HSV-1 and Alzheimer’s disease. Herpes simplex viruses are able to cause latent infections in neurons with periodic reactivations throughout life. No vaccine exists to prevent infection by the herpes simplex viruses [4].

The first step in infection of a host is attachment of viral glycoprotein to a host receptor. Five different herpes simplex glycoproteins may be involved in attachment to host cells [5]. Entry into the host cell is initiated by binding with heparan sulfate and seems to involve a complex process of sequential binding of viral proteins with other host proteins [6]. Once the viral DNA is in the host nucleus, genome expression occurs in differential order. Alpha genes enhance expression of beta genes for replication followed by structural gamma genes [7]. Assembled capsids typically bud through the nuclear membrane.

Acyclovir, acting as a nucleoside analog, has been a useful chemotherapeutic intervention for HSV-1 and 2 infections since it is activated by viral thymidine kinase [8]. However, resistance to acyclovir has been increasing and is particularly problematic among immunocompromised individuals [9]. It would be advantageous to find additional therapeutic agents that could curtail herpes infection. Natural products have long been recognized as potential sources for antiviral compounds [10]. *Panax ginseng* Meyer has served as a traditional Chinese medicine for centuries [11]. Extracts of ginseng have been described as having numerous benefits, including anti-inflammatory effects [12], prevention of obesity [13], and anti-cancer activity [14]. Red ginseng has been reported to inhibit a wide variety of viruses including respiratory (influenza, rhinoviruses), gastrointestinal (enteroviruses, rotaviruses, noroviruses), and hepatic (both hepatitis A and B viruses)[15]. As reviewed, [16], ginsenoside Rg3 reduced the severity of HSV-induced keratitis and decreased herpes plaque formation in cultured human amniotic cells.

Over 100 saponins or ginsenosides have been isolated from ginseng, which are generally divided into two groups, protopanaxadiols and protopanaxatriols [17]. Well known protopanaxadiols include Rb1, Rb2, Rb3, Rc, Rd, Rg3, Rh1, Rh2 and Rs1 while well known protopanaxatriols include Re, Rf, Rg1, Rg2 and Rh1. Some ginsenosides exist as R or S isoforms 20(R) or 20(S) depending on the position of the hydroxyl moiety at carbon 20 [18]. Of the ginsenosides evaluated in this study, Rg2, Rg3, Rh1 and Rh2 occur as isoforms.

In this study, the most prevalent ginsenosides found in natural (white) and steamed (red) ginseng [19, 20] were evaluated for their antiviral activity against HSV-1 and HSV-2. These included Rb1, Rb2, Rb3, Rc, Rd, Re, Rf, Rg1, 20(R)-Rg2, 20(S)-Rg2, 20(R)-Rg3, 20(S)-Rg3, 20(R)-Rh1, 20(S)-Rh1, 20(R)-Rh2 and 20(S)-Rh2. The 20(R)-Rg2, 20(S)-Rg2, 20(R)-Rg3, 20(S)-Rg3, 20(R)-Rh1, 20(S)-Rh1, 20(R)-Rh2 and 20(S)-Rh2 ginsenosides are conversion products that are generated when white ginseng is converted by steaming into red ginseng. We report that 20(S)-Rg3 demonstrated strong inhibitory activity against both herpes simplex viruses. Our results suggest that 20(S)-Rg3 affects some aspect associated with the interaction of the virus and host receptor proteins.

## Materials and Methods

### Cells

Vero cells (American Type Culture Collection (ATCC), USA, CCL-81) were maintained in medium 199 (Sigma Chemical Company, USA) that was supplemented with 8% fetal bovine serum (Invitrogen, USA), 1.25 µg/ml fungizone (Invitrogen) and penicillin-streptomycin, 1,000 U/ml and 1 mg/ml respectively (Sigma). Cells were passed weekly using 0.1% trypsin (Sigma) to detach the cells from the flasks. Cell quantification was done by trypan blue exclusion.

### Test Chemicals

**Fig. 1:**
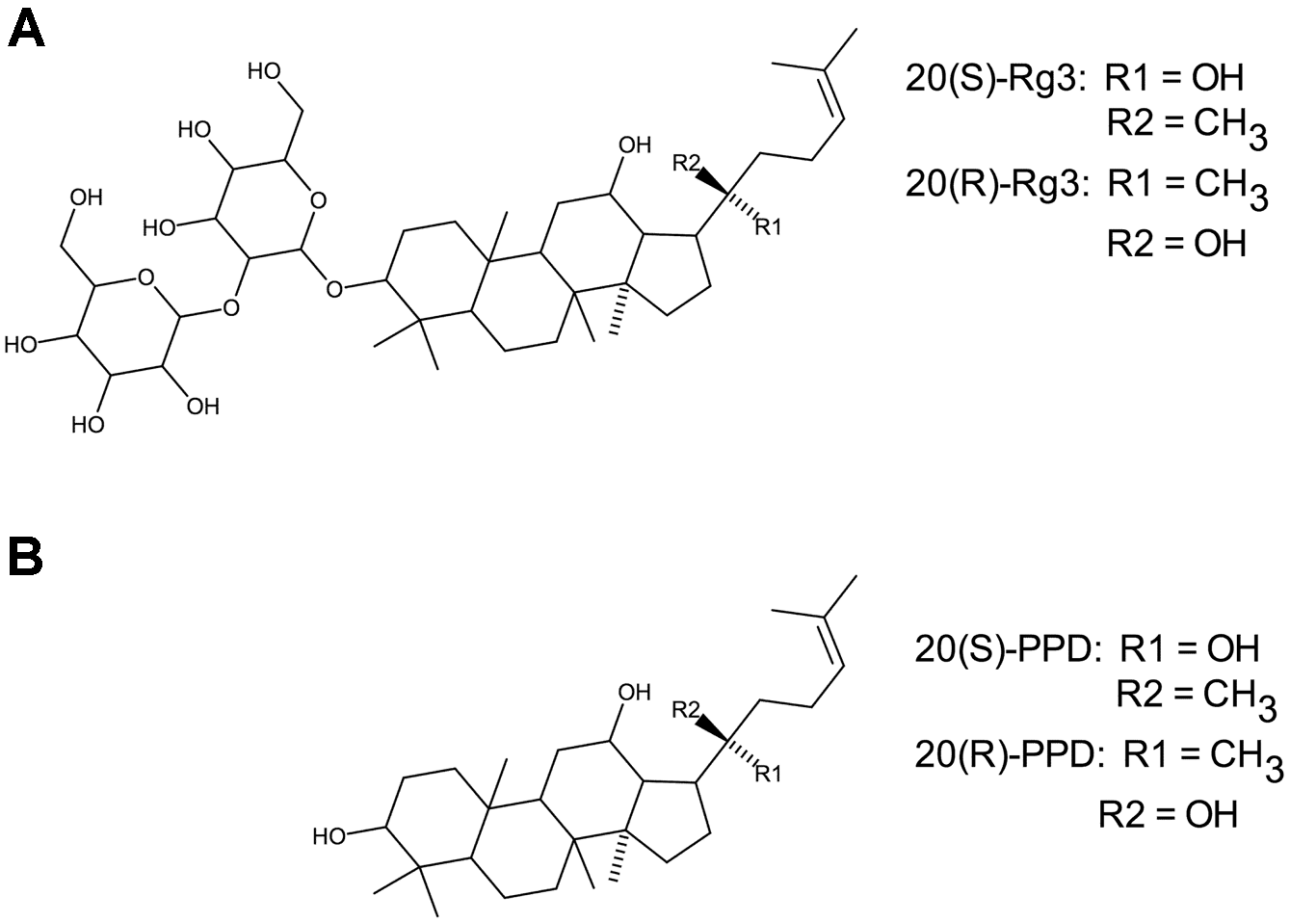
Structures of ginsenoside Rg3 and protopanaxadiol. (**A**) 20(S)- and 20(R)-Rg3 chemical structures are shown. (**B**) 20(S)- and 20(R)-protopanaxadiol (PPD) chemical structures are shown.

Twelve of the most prevalent ginsenosides, including the R and S isoforms for the four ginsenosides that had R and S confirmations, were evaluated for anti-herpes simplex activity. These included Rb1, Rb2, Rb3, Rc, Rd, Re, Rf, Rg1, 20(R)-Rg2, 20(S)-Rg2, 20(R)-Rg3, 20(S)-Rg3, 20(R)-Rh1, 20(S)-Rh1, 20(R)-Rh2 and 20(S)-Rh2. Additionally the ginsenoside metabolites 20(R)-protopanaxadiol and 20(S)-protopanaxadiol were also tested, because of the similarity in structure to 20(R)-Rg3 and 20(S)-Rg3, differing only by the lack of the disaccharide sophorose. These chemical structures are shown in Fig. 1. Structures of all ginsenosides evaluated in this study are provided as Supplemental Information, in Fig. S1. Valacyclovir (Sigma), a known anti-herpes compound, was used as a control for herpes inhibition. All compounds derived from *P. ginseng* were obtained from Biopurify Phytochemicals Ltd. at 98% purity (China) and were prepared in DMSO.

### Viruses and Quantification

Herpes simplex virus type 1 (HSV-1, MacIntyre strain, ATCC, VR-539) or herpes simplex virus type 2 (HSV-2, ATCC, VR-734) were used in these studies. Virus titer was determined by plaque assay. Briefly, following 2 h adsorption of various virus dilutions onto a Vero cell monolayer in a 24-well plate, the medium was removed and cells were overlaid with 1.5% carboxymethylcellulose (Sigma) and incubated 48 h at 37°C with 5% CO_2_. After incubation, cells were fixed with methanol, stained with Giemsa (Sigma) and plaques were counted. Each virus dilution was prepared in quadruplicate.

The plaque assay procedure also was used to evaluate the percent plaque reduction; the only procedural change was that a constant virus concentration was used (50 PFU/well). Plaque reduction was measured by comparing the control (virus only), with valacyclovir (16 µM) with virus or 20(S)-Rg3 (50 µM) with virus. Sample wells were evaluated in quadruplicate. Following the 2 h virus adsorption, the methylcellulose-medium containing the same concentrations of valacyclovir or 20(S)-Rg3 was added to the appropriate wells. After 48 h, plaques were fixed, stained and counted.

### Cytotoxicity and Virus Inhibition

All ginsenosides, isomers, and protopanaxadiols were evaluated for toxic effects on Vero cells prior to anti-viral testing. Cells were seeded at 5,000 cells/well in a black 96-well plate with clear bottom in a volume of 100 µl and allowed to attach for 24 h. The *P. ginseng* compounds were prepared in DMSO at 100 µM and were taken through serial two-fold dilutions to determine the concentration when the rate of cell death was less than 10%compared to control, untreated cells. Each dilution was tested in three wells and all assays were repeated three times. Following 48 h incubation at 37°C in 5% CO_2_, 11 µl of PrestoBlue (Invitrogen) was added to each well and the plate was incubated for an additional 30 min. PrestoBlue determines cell viability by changing from blue to red as resazurin is reduced to resorufin by viable cells. Following the PrestoBlue incubation, fluorescence was determined by spectrophotometric reading using Soft Max Pro (Molecular Diagnostics, USA).

For cytotoxicity testing, 4 µl of dilutions of the ginsenosides or protopanaxadiols were added to 396 µl of medium 199 and 100 µl of this mixture was added to each of three wells. For anti-viral testing, 4 µl of the non-toxic reagent and 4 µl of HSV-1 or HSV-2 diluted to an MOI of 0.1 were added to 392 µl of medium 199 and 100 µl of this was added to each of three wells. Virus inhibition was determined by comparing the cell death of reagent-exposed cells with cell death of control cells only receiving virus. The 50%inhibitory concentration (IC_50_) was determined through a series of two-fold dilutions and calculation by linear regression analysis.

### Time Studies

For virus inhibition studies described above, reagent 20(S)-Rg3 and virus were added to cells simultaneously. In an attempt to evaluate the potential mode of action of 20(S)-Rg3, various combinations of valacyclovir, 20(S)-Rg3 and HSV-1 were tested at different time periods. The following combinations were evaluated at both one hour and four hours:

● Virus applied to cells first (for one hour or four hours), followed by addition of valacyclovir, 20(S)-Rg3 or both valacyclovir and 20(S)-Rg3 to the cells
● Valacyclovir or 20(S)-Rg3 or both valacyclovir and 20(S)-Rg3 applied to cells first (for one or four hours), followed by virus
● Valacyclovir or 20(S)-Rg3 incubated together with virus first for one or four hours then added to cells

All procedures described for evaluation of virus inhibition were followed in identical fashion as outlined for the PrestoBlue procedure except valacyclovir was used at a constant concentration of 16 µM and 20(S)-Rg3 at 50 µM. The use of valacyclovir at 16 µM, or 5.76 µg/ml, is in the reported IC_50_ ranges for this drug, from 0.02-13.5 µg/ml (New Drug Application #20-487/S-007, GlaxoSmithKline, available from https://www.accessdata.fda.gov/drugsatfda_docs/label/2005/020487S007rel2_lbl.pdf).

### Statistical Analysis

All values are shown as the mean ± standard error. A one-tailed Student’s *t*-test was used to determine significance between groups. The level for significance was *p* < 0.05.

## Results

### Toxicity

All ginsenosides and the closely-related metabolites 20(R) and 20(S)-protopanaxadiol were tested for toxic effects on Vero cells. Both protopanaxadiol isoforms were the most toxic compounds and had to be diluted to 12.5 μM to cause less than 10 percent cell death compared to controls (Table 1). The majority of compounds tested resulted in less than 10% cell death when used at the highest starting concentration of 100 μM. Since 20(S)-Rg3 was determined to effectively inhibit herpes simplex viruses, additional testing was done to find the actual concentration that was toxic to Vero cells, starting at 1,000 μM and making 2-fold dilutions (Table 2). When used at 1,000 μM, 20(S)-Rg3 resulted in less than 1% viable cells. Less than 20% of cells remained viable when Rg3 was used at 500 μM. Subsequent two-fold dilutions were non-toxic and at concentrations of 250 μM and less, exposure to 20(S)-Rg3 had a proliferative effect on cells or at least enhanced metabolic activity compared to untreated cells. The non-toxic concentration of all compounds shown in Table 1 was used for virus inhibition tests. The known herpes simplex inhibitor valacyclovir was used at 16 μM; cells retained nearly 99% viability at this concentration (1.2 ± 0.4 percent cell death occurred).

### Virus Inhibition

For all ginsenosides, isoforms, or metabolites, only 20(S)- Rg3 was able to dramatically inhibit HSV-1 (Table 1). While 20(S)-Rh2 inhibited HSV-1 by 46.2%, it was approximately half as effective as 20(S)-Rg3. Given the effectiveness of 20(S)-Rg3, additional investigations were carried out with this molecule. When coupled with virus, one of the ginsenosides, Rf, and the metabolite 20(S)-protopanaxadiol resulted in greater cell death than virus alone, even when used at a non-toxic level.

**Fig. 2:**
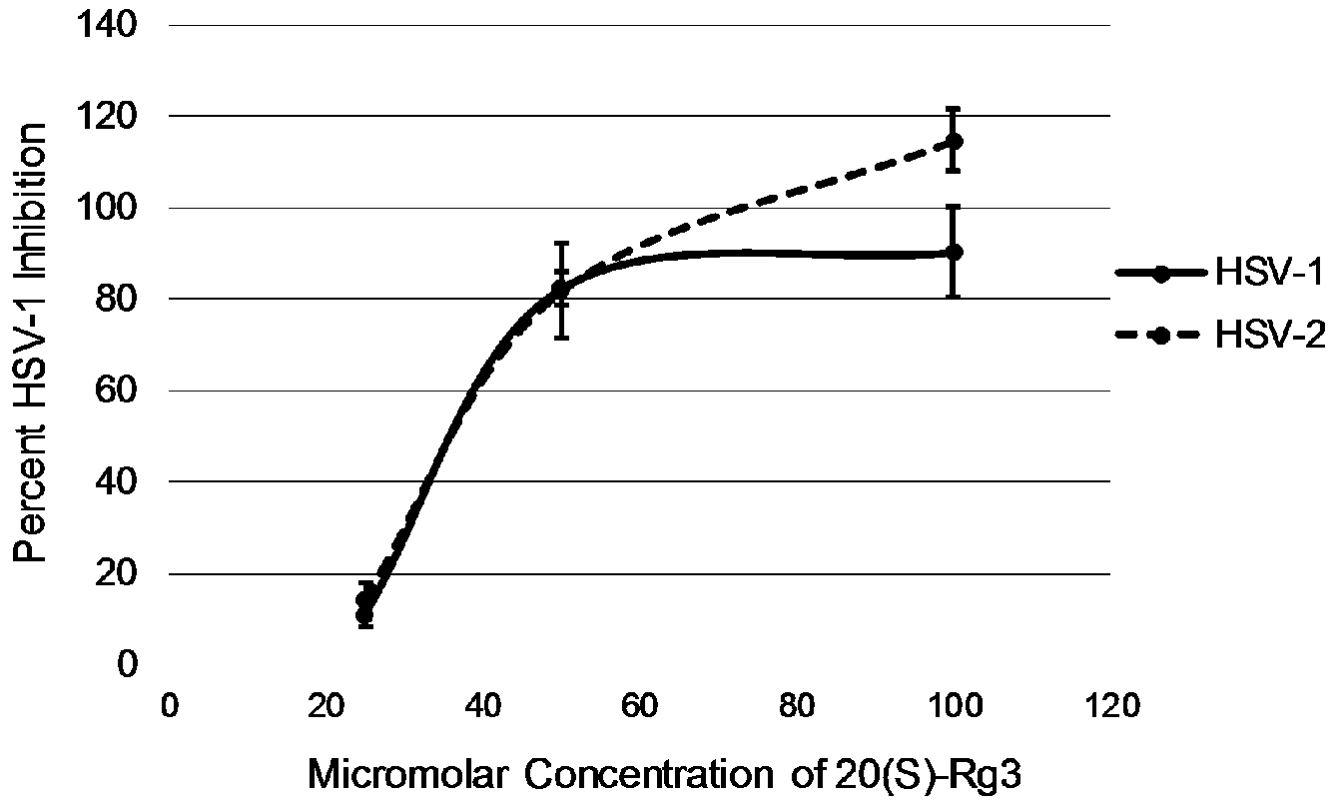
Dose response curve for 20(S)-Rg3. For both HSV-1 and HSV-2, the IC_50_ was approximately 35 μM.

Due to the effective antiviral activity of 20(S)-Rg3 against HSV-1, 20(S)-Rg3 was also evaluated for inhibition of HSV- 2. As evident in Table 3, both of these herpes alphaviruses were nearly completely inhibited when 20(S)-Rg3 was used at 100 μM. At 50 μM, each virus was suppressed by 80%. The IC_50_ of 20(S)-Rg3 was determined to be approximately 35 μM as shown in Fig. 2. As a control, valacyclovir had an IC_50_ concentration of 13.2 μM against HSV-1.

Plaque reduction of HSV-1 by 20(S)-Rg3 was also tested to evaluate bioactivity. Table 4 shows the percent reduction of HSV-1 by valacyclovir (used at 16 μM) and 20(S)-Rg3 (50 μM). All wells were infected with the same concentration of virus (approximately 50 PFU each) but only 1.5 ± 0.3 plaques were visible on cells treated with 20(S)-Rg3.

### Time Studies

**Fig. 3:**
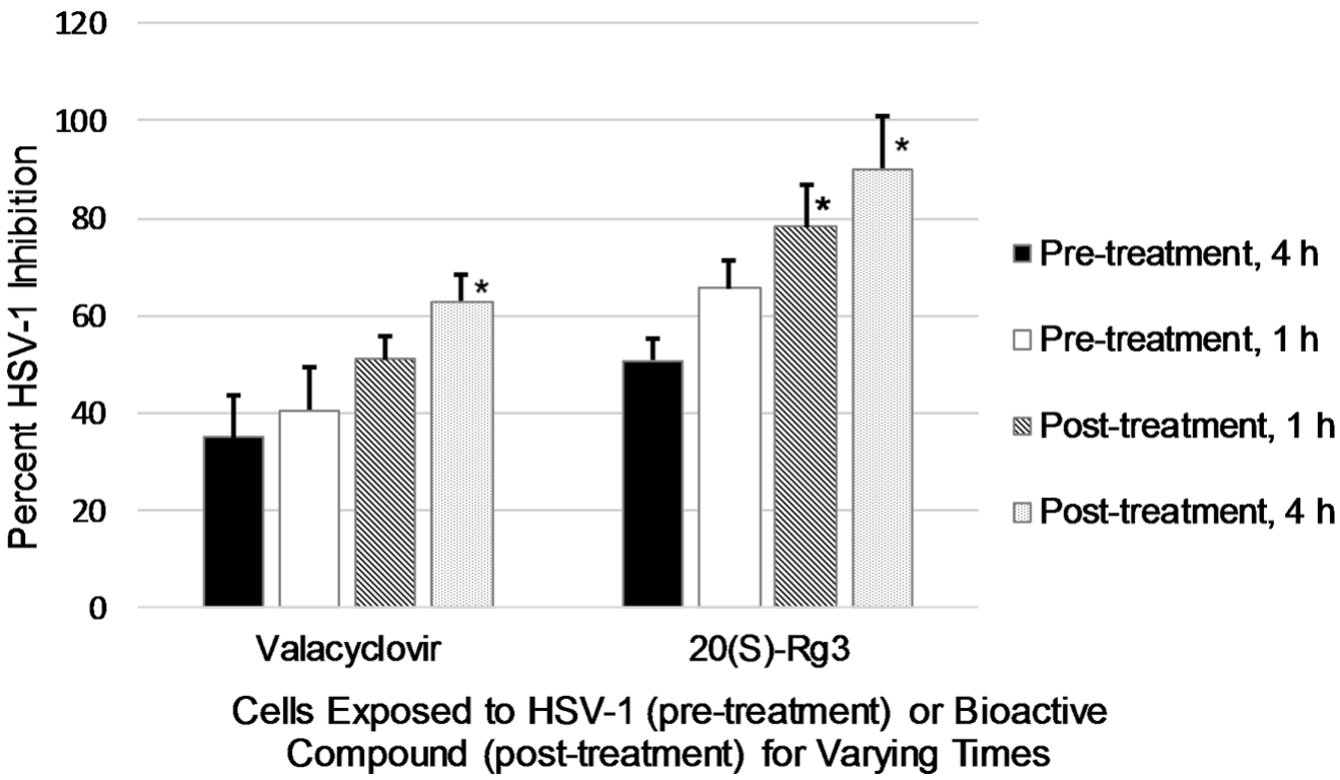
Percent HSV-1 inhibition. Cells were exposed to virus before the addition of bioactive compounds (pre-treatment; 4 h = black bars, 1 h = white bars) or exposed to bioactive compounds prior to virus being added (posttreatment; 4 h = stippled bars, 1 h = hatched bars). The asterisks indicate significant differences between 4 h pre-treatment values and post-treatment, *p* < 0.05.

The length of time for exposure of cells to virus or to bioactive compound was tested. As shown in Fig. 3, when cells were exposed to HSV-1 before treatment with either valacyclovir (16 μM) or 20(S)-Rg3 (50 μM), virus inhibition was reduced. When virus was present on cells 4 h prior to 20(S)-Rg3 addition, the percent inhibition was only 50.7 ± 4.4. When cells were treated with 20(S)-Rg3 prior to HSV-1 addition, the virus was inhibited more effectively. As the length of time for bioactive reagent pretreatment increased to 4 h, virus inhibition also increased, reaching a maximum percent inhibition of 90.1 ± 11.1 for 20(S)-Rg3. Post-treatment with 20(S)-Rg3 at both 1 and 4 h resulted in significantly increased virus inhibition (*p* < 0.05) compared to pre-treatment with virus for 4 h.

**Fig. 4:**
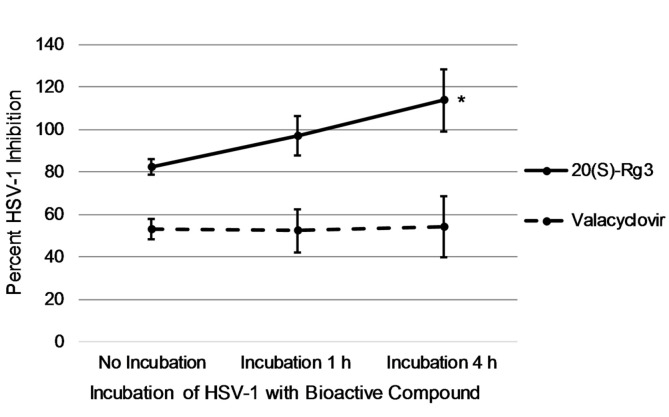
Percent inhibition of HSV-1 following incubation of virus with bioactive compounds. HSV-1 and the bioactive compound, valacyclovir or (20)S-Rg3, were applied directly to cells simultaneously (no incubation) or the virus and compound were incubated together for 1 h or 4 h prior to being added to the cells. A significant difference exists between the incubation of HSV-1 and 20(S)-Rg3 for 4 h compared with no incubation, *p* < 0.05. No differences existed for incubation of HSV-1 and valacyclovir.

When HSV-1 was incubated together with valacyclovir prior to addition to cells, there was no change in virus inhibition, ranging from 52.3 to 54.7 percent from 0 to 4 h (Fig. 4). However, as incubation of HSV-1 with 20(S)-Rg3 increased to 4 h, there was a dramatic increase in inhibition of virus to 113.7 ± 14.8 percent (*p* < 0.05). When virus was incubated together 4 h with 20(S)-Rg3 and valacyclovir simultaneously, a synergistic effect was evident with percent virus inhibition at 125.3 ± 4.9.

## Discussion

In this study the 12 most prevalent ginsenosides from natural (white) or steamed (red) ginseng were examined as potential inhibitors of HSV-1 and HSV-2. 20(S)-Rg3 demonstrated significant antiviral activity with an IC_50_ of approximately 35 μM against both HSV-1 and HSV-2. Additionally, 20(S)-Rg3 had very low toxicity, with an IC_50_ value of approximately 400 μM against the host Vero cells. With a selectivity of 11-fold, 20(S)-Rg-3 appears to be very promising as an anti-viral agent against HSV-1 and HSV-2.

The ginsenoside metabolite 20(S)-protopanaxadiol has been reported to have pharmacological activity [21] and is structurally similar to ginsenoside 20(S)-Rg3. Thus it seemed prudent to include protopanaxadiol in toxicity and anti-herpes investigations. The concentration of 20(S)-protopanaxadiol found to be non-toxic in this study was 12.5 μM which is in agreement with Bak [22] who determined that a concentration of 20 μM was not toxic for PC12 cells. Protopanaxadiol has been found to be an effective anti-oxidant that preserves healthy mitochondrial function [22] as well as providing protection for UV-associated skin wrinkling [21]. However, in this study, both protopanaxadiol isoforms failed to have any protective effect against HSV-1.

Several ginsensoides, both protopanaxadiols and protopanaxatriols, have been reported to have anti-viral effects. Rb1 decreased the apoptotic effects of HSV-1 in a human glioma cell line [23]. In that study, to achieve inhibition of herpes a concentration of 400 μg/ml was necessary. We found that Rb1 caused greater than 10%Vero cell death when used at a concentration higher than 50 μM and that minimal inhibition (23%) of HSV-1 occurred at this non-toxic concentration. Chen *et al*. reported [24] that Rh2 suppressed tumor growth in hepatitis B virus hepatocellular carcinoma cells. The protopanaxatriols Re, Rf, and Rg2 had inhibitory effects on coxsackie B3 virus and human rhinovirus 3 at 100 μg/ml [25]. Ginsenoside-Re has been reported to have anti-influenza virus activity due to enhancing immune responses after infection in mice [26]. None of these ginsenosides exhibited at least 50% inhibition of HSV-1 in our investigation.

One of the bioactive properties attributed to Rg3 includes anti-cancer effects by decreasing drug efflux from a cancer cell line [27]. In that study, Rg3 was cytotoxic for human carcinoma cells KB V20C when used at 120 μM but this concentration had no effect on normal WI 38 cells. Rg3 has been determined to have potent anti-inflammatory activity by down-regulation of cytokine expression [28]. Rg3 has also been recognized to inhibit several virus life cycles. 20(S)-Rg3, but not 20(R)-Rg3, was reported to prevent rotavirus infection in mice, presumably due to mediation of immune responses [29]. Studies have shown that Rg3 reduces the expression of epidermal growth factor receptor on cells [30]. Such down-regulation of host receptors may impact viral attachment to a potential host. Kim [14] described Rg3 as a potential therapeutic agent for hepatitis C virus. Rg3 restored normal mitochondrial processes that hepatitis C had disrupted, thus helping prevent chronic virus infection. Rg3 also has been reported to inhibit hepatitis B virus capsid maturation by interfering with pro-inflammatory cytokine expression [31]. Kang *et al*. [32] determined that Rg3 prevented the replication of a murine gammaherpes virus, likely through inhibition of cellular signaling pathways. A crude extract of Korean red ginseng has been documented to inhibit HSV-1 vaginal infection in mice [33]. The mechanism for the protective effects of the ginseng extract was suggested to involve stimulation of immune responses, particularly interferon γ and natural killer cells. In the current study, purified 20(S)-Rg3 was shown to effectively curtail replication by both HSV-1 and HSV-2. Decreased host cell death and plaque reduction by 20(S)-Rg3 indicate reduced success for completion of the herpes life cycle.

What may 20(S)-Rg3 be doing to virus-host interaction?The experiments shown in Fig. 3 demonstrated the therapeutic effects of 20(S)-Rg3, as well as valacyclovir, were strongest when cells were treated with the bioactive compounds before virus had an opportunity to attach and penetrate its host. This is in line with the known mechanism of valacyclovir as a nucleoside analog. Valacyclovir enters cells but remains inactive until HSV is also present. Increased anti-viral activity is seen with pre-exposure of cells to valacyclovir; upon viral infection, valacyclovir is already present, “waiting” to be activated. In relation to 20(S)-Rg3, while all aspects of herpes simplex attachment and penetration of host cells have not been fully elucidated, a good picture of the process has been described. Host receptor heparan sulfate is bound by herpes glycoproteins gB and gC [34]. The next step involves binding of gD and the glycoprotein dimer gH-gL with the Herpes Virus Entry Mediator receptors and nectin-1 or nectin-2, which are cell adhesion molecules [6]. In suggesting the effects of 20(S)-Rg3, the data from Fig. 3 allow a possible contributing mechanism for this ginsenoside. It has been reported that exposure of endothelial cells to Rg3 resulted in decreased expression of adhesion molecules, both vascular cell adhesion molecule-1 and intracellular cell adhesion molecule-1 [28]. Optimal receptors for HSV attachment include the cell adhesion molecules nectin-1 and nectin-2. Further, Uchida and colleagues [35] investigated HSV-1 attachment and penetration using mutations in gB and gD. When a mutation in gD prevented normal binding with the Herpes Virus Entry Mediator, the virus was still able to penetrate the host cell through an epidermal growth factor receptor. Thus, a possible mechanism of 20(S)-Rg3 inhibition of herpes simplex viruses may relate to binding with such alternate receptors since Rg3 has been reported to down-regulate epidermal growth factor receptor [30].

A second possible mechanism of 20(S)-Rg3 is suggested by the experiments shown in Fig. 4, where the virus was incubated with the bioactive compounds prior to exposure to host cells. For valacyclovir, there was no difference in anti-viral activity whether or not virus was exposed to valacyclovir prior to infecting cells. This is expected since valacyclovir is only activated by viral thymidine kinase once the virus is replicating inside the host cell. However, when 20(S)-Rg3 was allowed to interact for 4 h with the virus prior to exposure to host cells, successful production of virus was reduced significantly over 110%. Given the array of at least five possible attachment glycoproteins, it is reasonable to hypothesize that incubation of HSV-1 with 20(S)-Rg3 prior to exposure to host cells may result in ginsenoside binding with a viral attachment protein, decreasing the opportunity for viral penetration. While the possible impact of 20(S)-Rg3 on viral entrance to host cells is speculative, there is little doubt that 20(S)-Rg3 has a pronounced effect on the successful completion of the viral life cycle, particularly evident by the dramatic reduction in PFUs (Table 4).

Another promising aspect for chemotherapeutic use of 20(S)-Rg3 is suggested by the synergistic effect that occurred when the ginsenoside was used together with valacyclovir, inhibiting virus activity by 125%. Using two pharmacological agents with differing modes of action would not only be effective at inhibiting herpes simplex virus, it would also be useful in combating increasing resistance to acyclovir.

## Supplemental Materials



Supplementary data for this paper are available on-line only at http://jmb.or.kr.

## Figures and Tables

**Table 1:** Cytotoxicity and inhibition of HSV-1 by ginsenosides and protopanaxadiols.

Ginsenosides & Protopanaxadiols	Concentration for Cytotoxicity Testing (μM)	Percent Inhibition of HSV-1
Rb1	50	23.3
Rb2	100	26.4
Rb3	100	30.9
Rc	100	28.6
Rd	100	36.1
Re	100	26.7
Rf	100	(38.0) ^a^
Rg1	100	13.2
20(S)-Rg2	100	44.3
20(R)-Rg2	100	25.3
20(S)-Rg3	100	90.3
20(R)-Rg3	100	22.7
20(S)-Rh1	100	14.2
20(R)-Rh1	100	26.3
20(S)-Rh2	25	46.2
20(R)-Rh2	50	12.0
20(S)-protopanaxadiol	12.5	(1.7)
20(R)-protopanaxadiol	12.5	14.4

The non-toxic concentration was defined as causing less than 10% cell death compared with controls and was used to determine virus inhibition. To determine cytotoxicity, all compounds were initially tested starting at 100 μM. Subsequent 2-fold dilutions were made as necessary.

^a^A value in parentheses indicates that the non-toxic concentration with virus resulted in a higher percentage of cell death than the control that was exposed to the virus alone.

**Table 2:** Cytotoxicity of 20(S)-Rg3 against Vero cells.

Concentration (μM)	Percent Cell Viability
50	(113.0 ± 3.1) ^a^
100	(114.3 ± 4.5)
125	(122.3 ± 1.8)
250	(128.7 ± 1.4)
500	17.7 ± 14.7
1,000	0.4 ± 0.02

^a^Values in parenthesis indicate greater cell proliferation or metabolic activity compared to untreated control cells.

**Table 3:** Inhibition of herpes simplex viruses by 20(S)-Rg3.

20(S)-Rg3 Concentration μM	Percent Inhibition of HSV-1	Percent Inhibition of HSV-2
25	11.0 ± 2.6	14.3 ± 3.3
50	82.3 ± 3.5	81.7 ± 10.3
100	90.3 ± 9.9	114.7 ± 6.7

The values are the percent inhibition ± the standard error compared to the control cells that were exposed to the viruses but not to 20(S)-Rg3.

**Table 4:** Plaque reduction of HSV-1.

	Number of Plaques	Percent Reduction
Control	56.7 ± 1.2	
Valacyclovir	8.1 ± 1.1	85.7
20(S)-Rg3	1.5 ± 0.3	97.3

The number of plaques ± the standard error and the percent reduction compared to the control after treatment by valacyclovir or 20(S)-Rg3 are shown.
