# Supplementary material for: Inhibition of Herpes Simplex Viruses, Types 1 and 2, by Ginsenoside 20(S)-Rg3
Source: J Microbiol Biotechnol. 2019 Nov 6;30(1):101–8. doi: 10.4014/jmb.1908.08047 (PMC9728284; doi:10.4014/jmb.1908.08047)
Supplement: Supplementary file 1 [file JMB-30-1-101-supple.pdf]

J. Microbiol. Biotechnol.
https://doi.org/10.4014/jmb.1908.08047

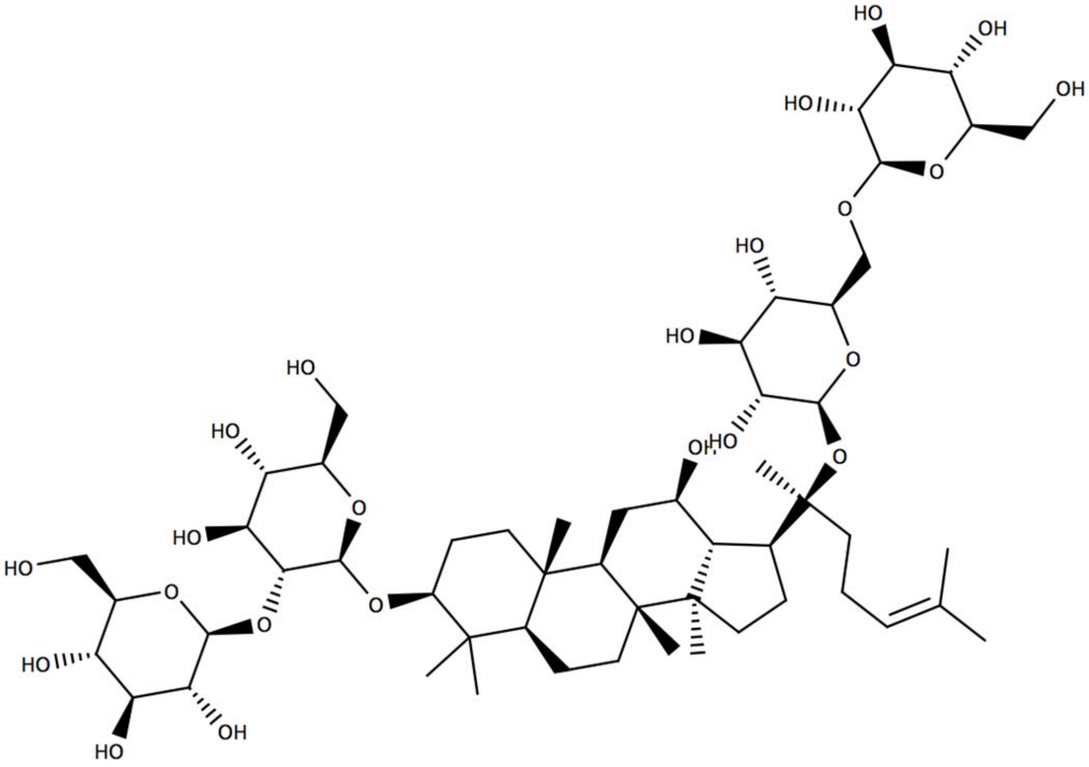

SFigure 1a. Ginsenoside Rb1.

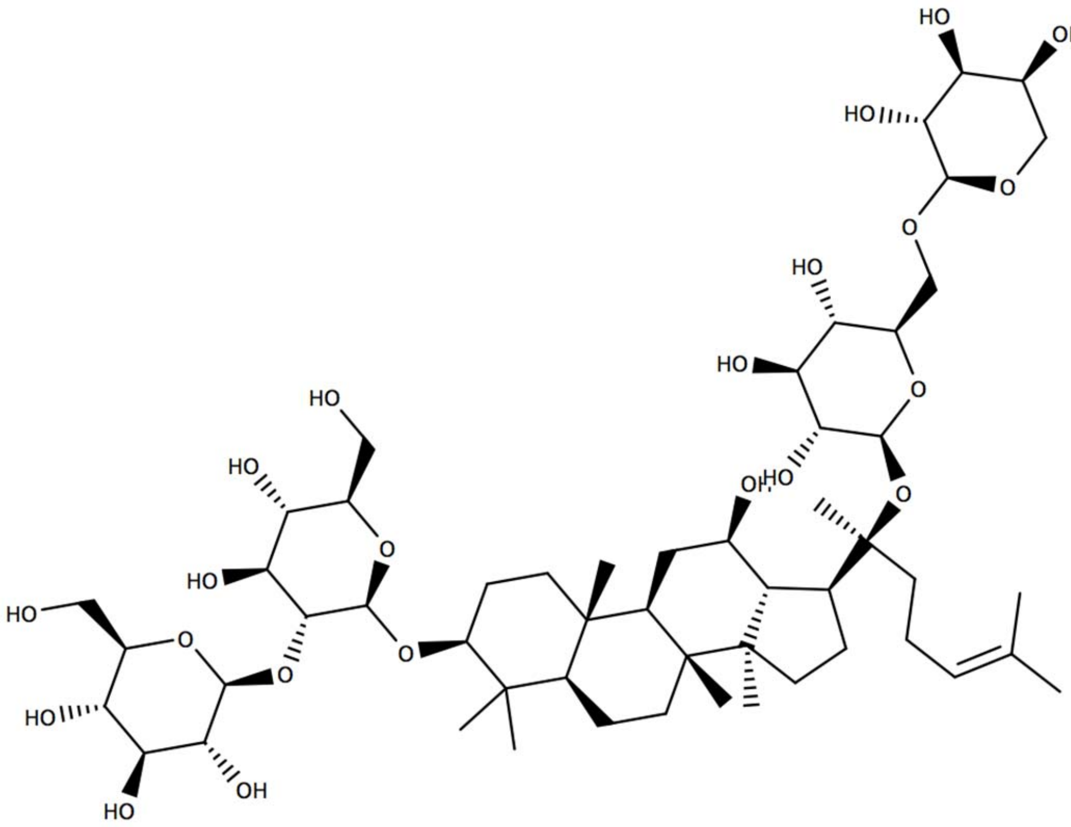

SFigure 1b. Ginsenoside Rb2.

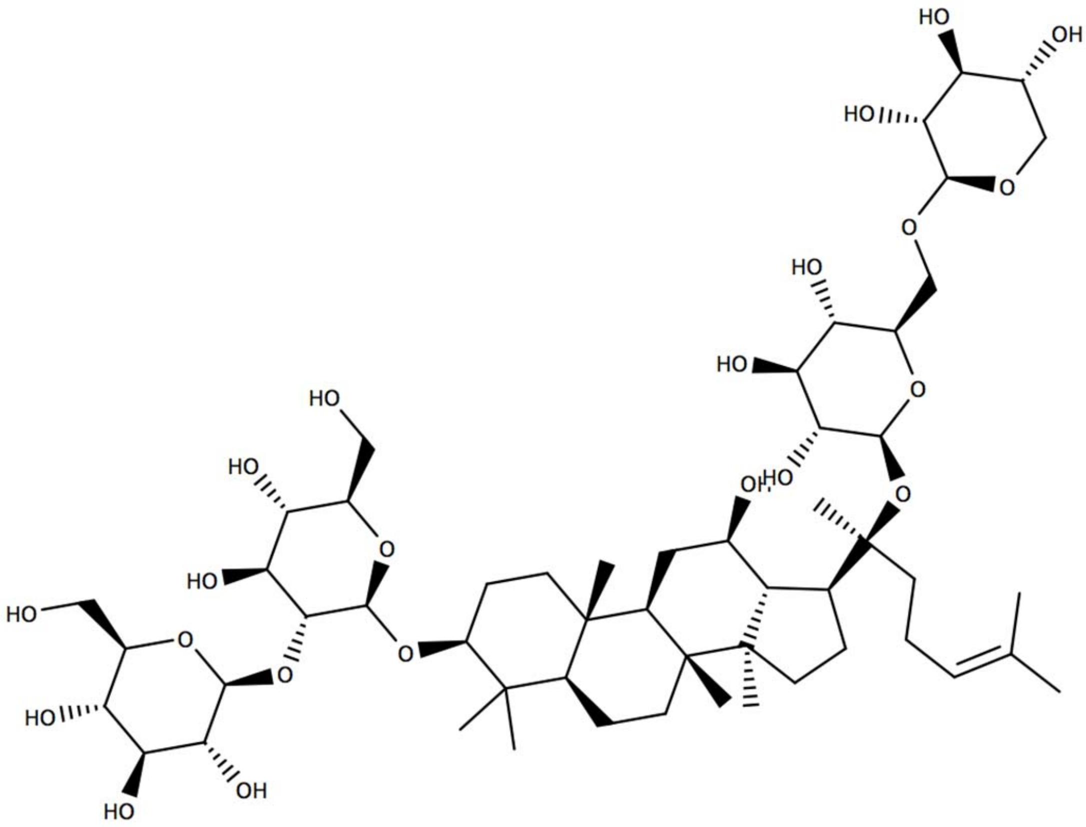

SFigure 1c. Ginsenoside Rb3.

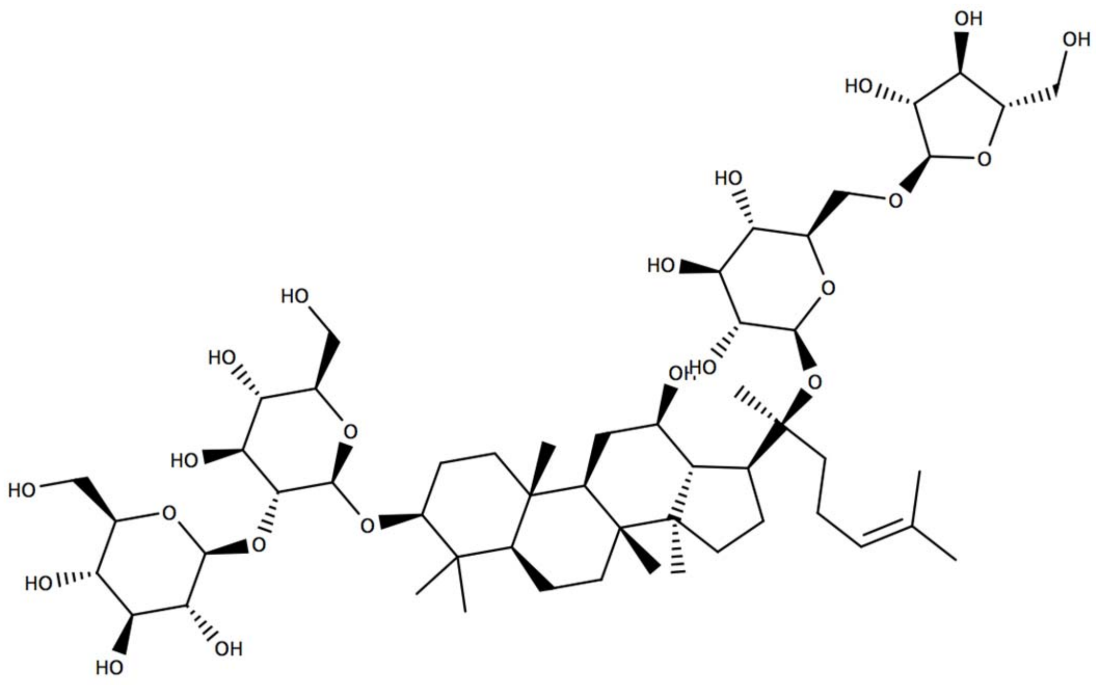

SFigure 1d. Ginsenoside Rc.

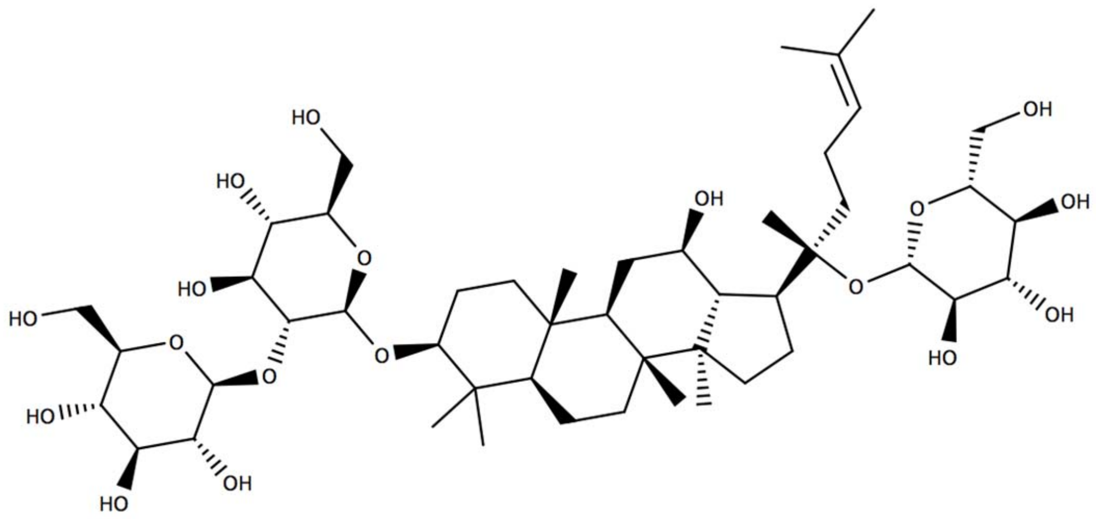

SFigure 1e. Ginsenoside Rd.

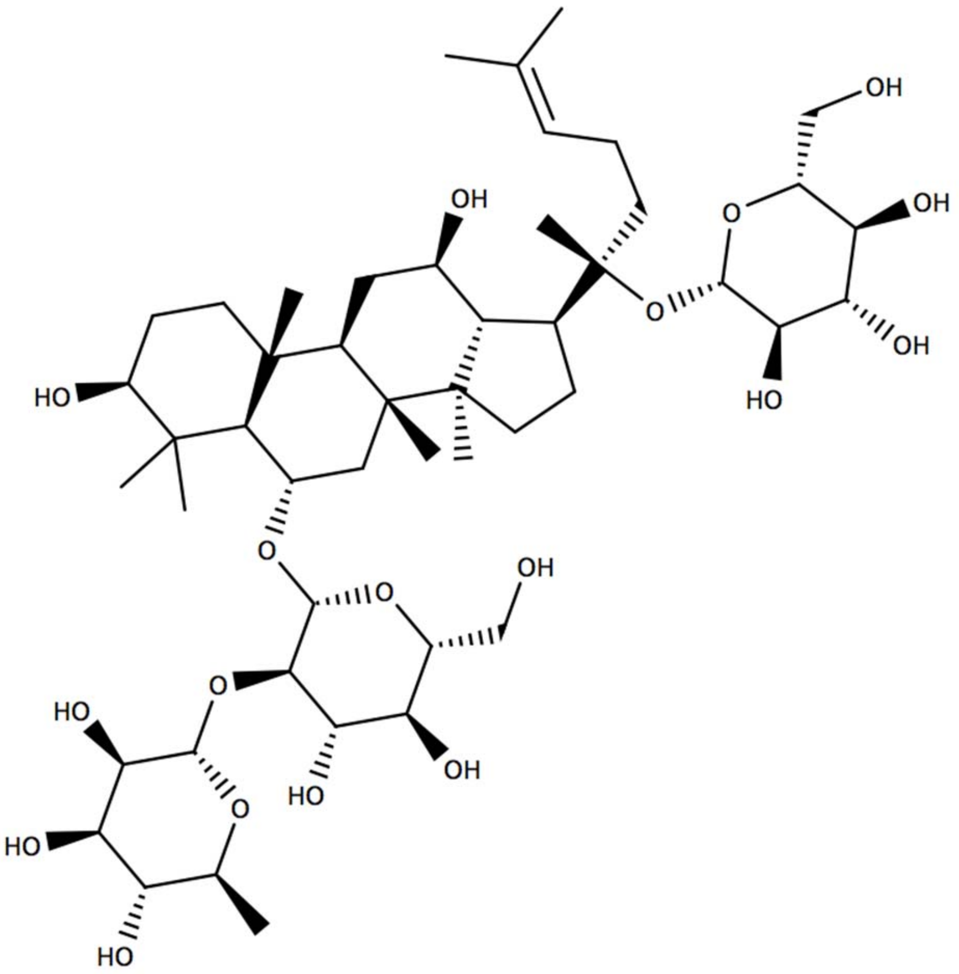

SFigure 1f. Ginsenoside Re.

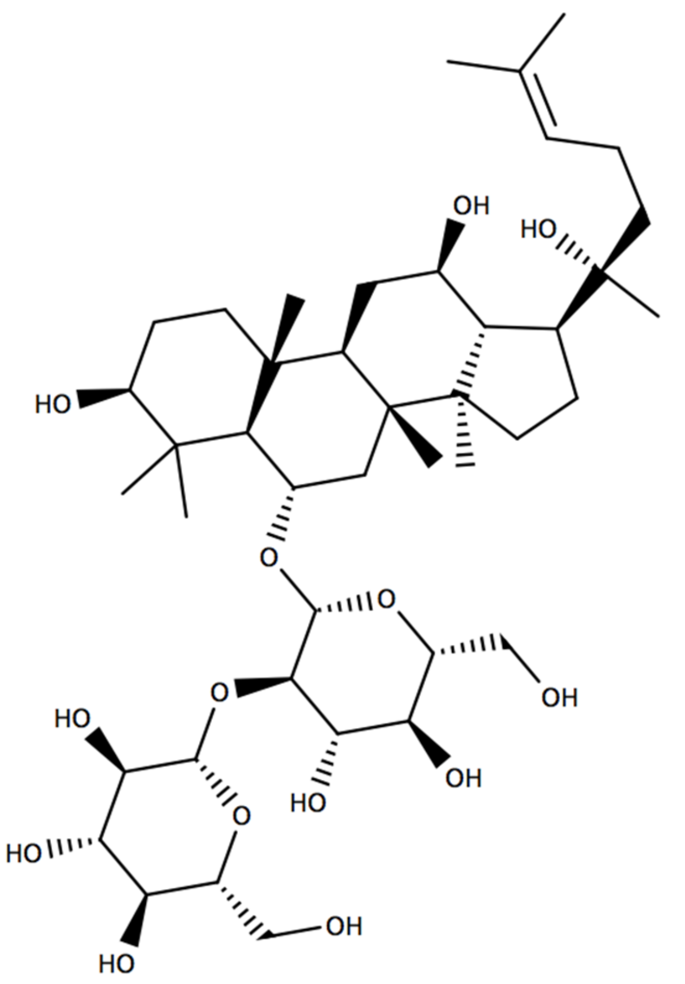

SFigure 1g. Ginsenoside Rf.

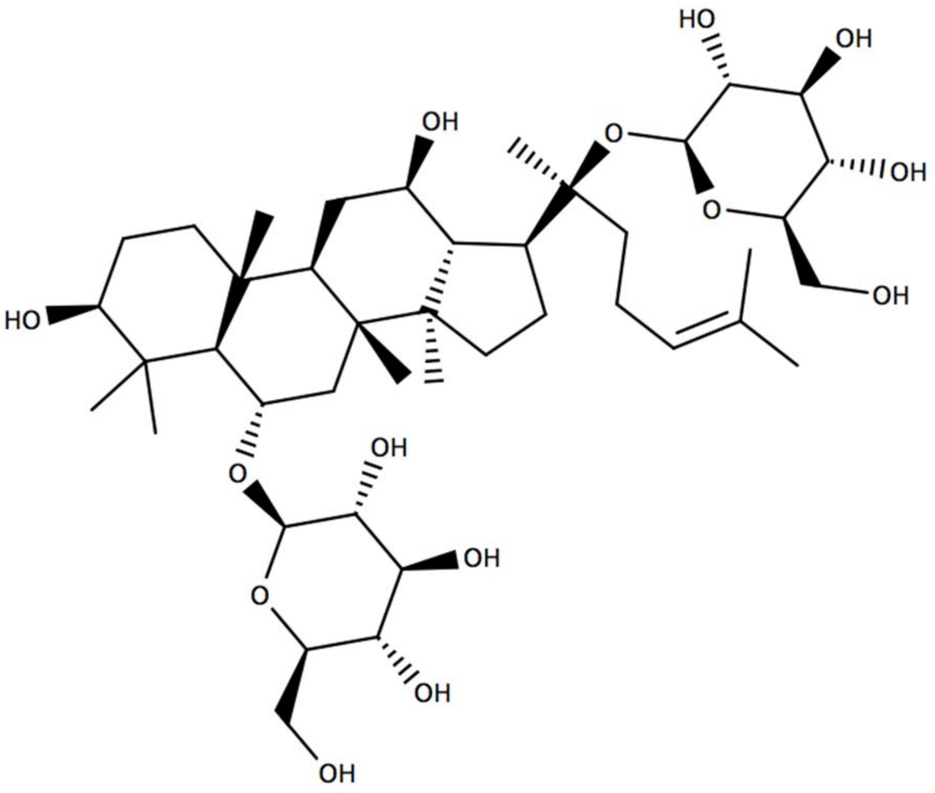

SFigure 1h. Ginsenoside Rg1.

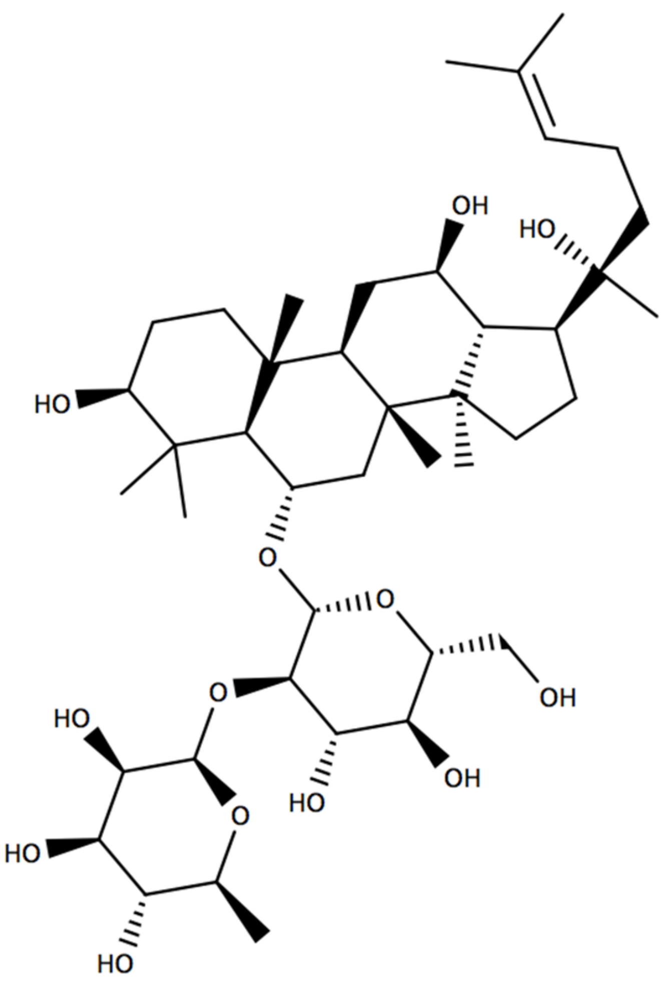

SFigure 1i. 20(S)-Ginsenoside Rg2.

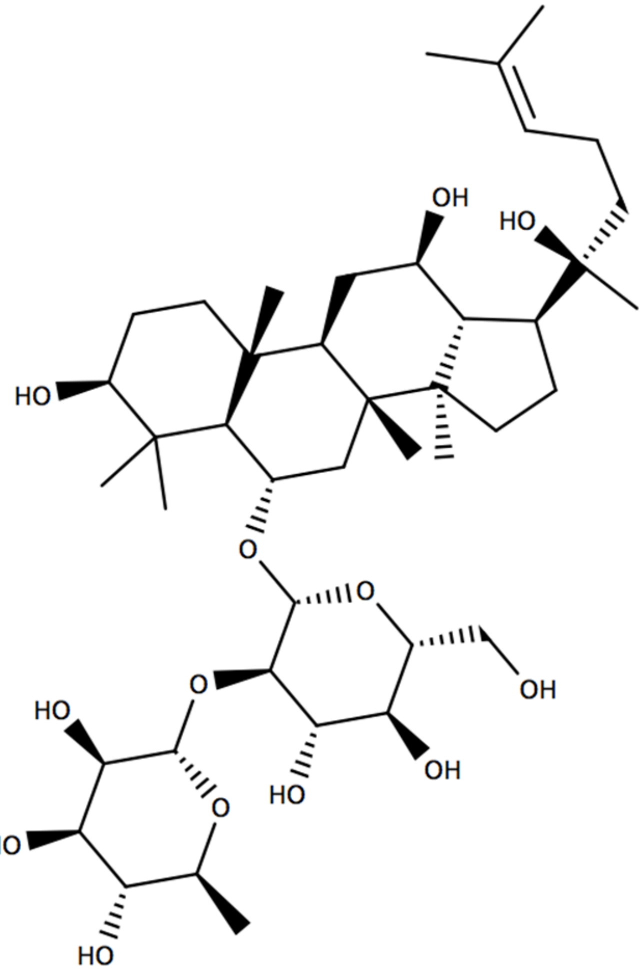

SFigure 1j. 20(R)-Ginsenoside Rg2.

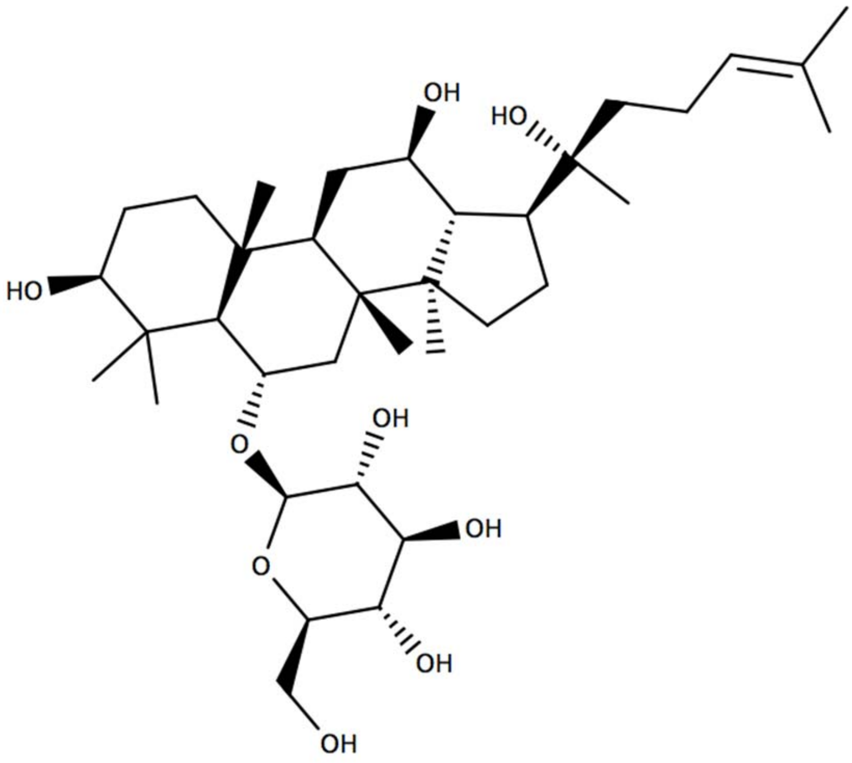

SFigure 1k. 20(S)-Ginsenoside Rh1.

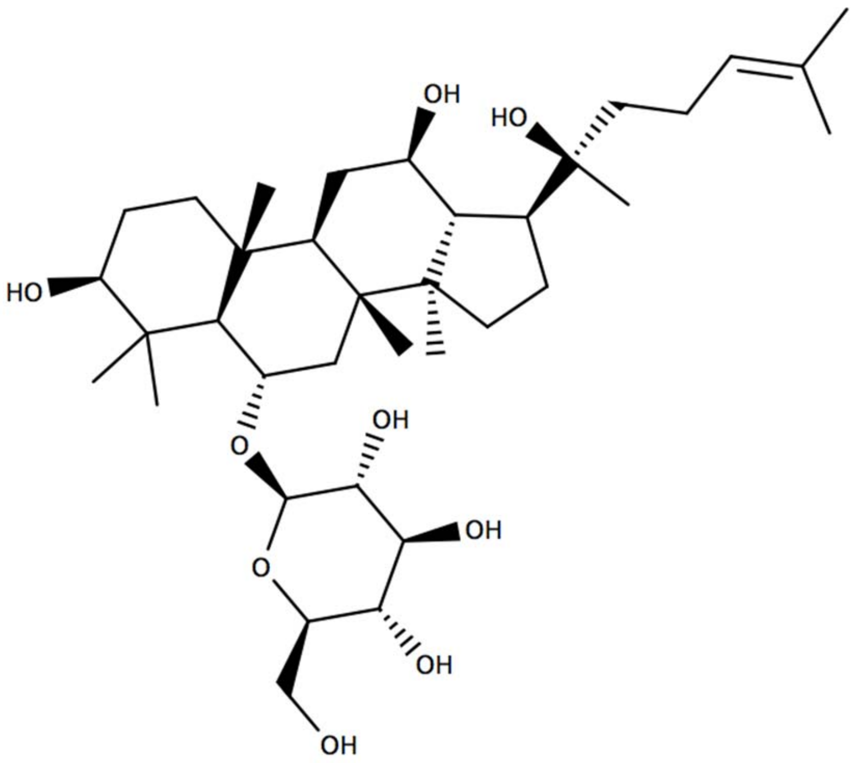

SFigure 1l. 20(R)-Ginsenoside Rh1.

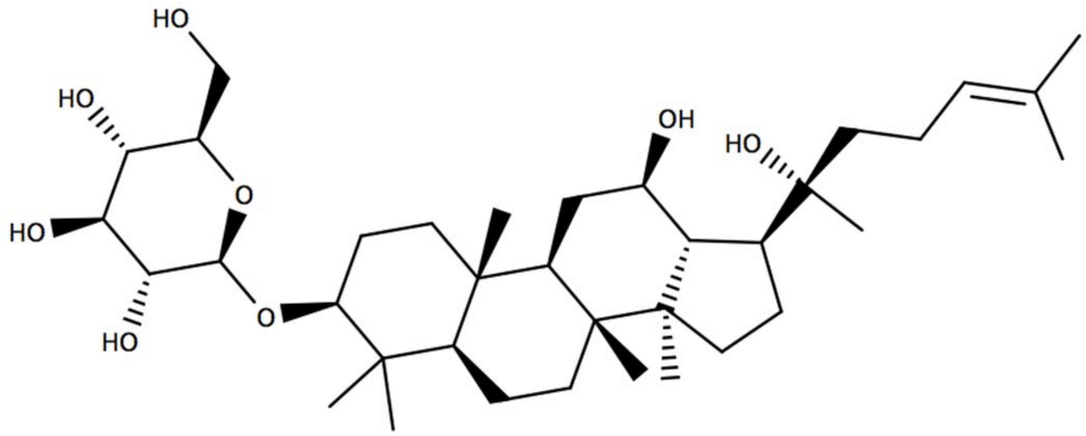

SFigure 1m. 20(S)-Ginsenoside Rh2.

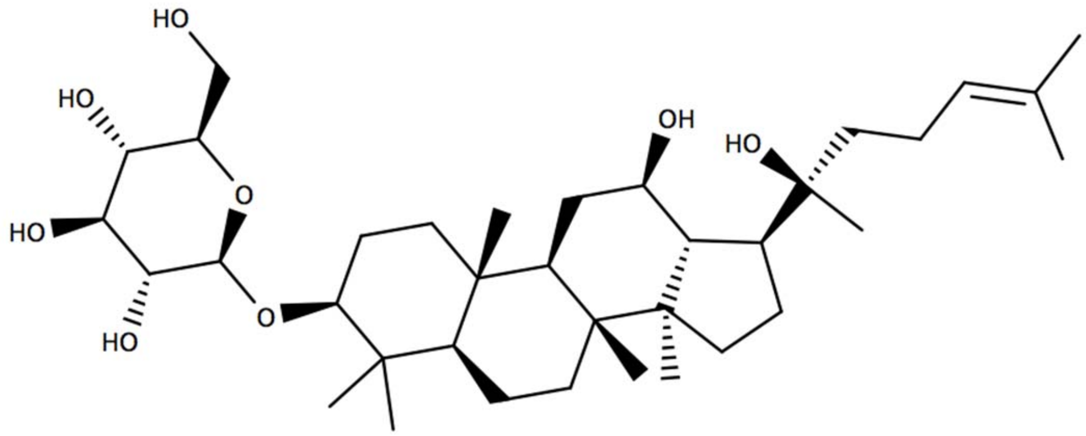

SFigure 1n. 20(R)-Ginsenoside Rh2.
